# Three Structural Features of Functional Food Components and Herbal Medicine with Amyloid β42 Anti-Aggregation Properties

**DOI:** 10.3390/molecules24112125

**Published:** 2019-06-05

**Authors:** Kazuma Murakami, Kazuhiro Irie

**Affiliations:** Division of Food Science and Biotechnology, Graduate School of Agriculture, Kyoto University, Kyoto 606-8502, Japan

**Keywords:** amyloid β, Alzheimer’s disease, aggregation, oligomer, neurotoxicity, natural product, flavonoid, triterpenoid, NMR, mass spectrometry

## Abstract

Aggregation of amyloid β42 (Aβ42) is one of the hallmarks of Alzheimer’s disease (AD). There are numerous naturally occurring products that suppress the aggregation of Aβ42, but the underlying mechanisms remain to be elucidated. Based on NMR and MS spectroscopic analysis, we propose three structural characteristics found in natural products required for the suppressive activity against Aβ42 aggregation (i.e., oligomerization by targeting specific amino acid residues on this protein). These characteristics include (1) catechol-type flavonoids that can form Michael adducts with the side chains of Lys16 and 28 in monomeric Aβ42 through flavonoid autoxidation; (2) non-catechol-type flavonoids with planarity due to α,β-unsaturated carbonyl groups that can interact with the intermolecular β-sheet region in Aβ42 aggregates, especially aromatic rings such as those of Phe19 and 20; and (3) carboxy acid derivatives with triterpenoid or anthraquinoid that can generate a salt bridge with basic amino acid residues such as Lys16 and 28 in the Aβ42 dimer or trimer. Here, we summarize the recent body of knowledge concerning amyloidogenic inhibitors, particularly in functional food components and *Kampo* medicine, and discuss their application in the treatment and prevention of AD.

## 1. Introduction

Alzheimer’s disease (AD) is an irreversible neurodegenerative disorder. The two pathological hallmarks of AD are the accumulation of amyloid β (Aβ) protein deposits in extracellular senile plaques and the accumulation of tau protein induced from hyperphosphorylation mainly by glycogen synthase kinase 3 β (GSK-3β) in intracellular neurofibrillary tangles [1]. The amyloid cascade hypothesis, originally proposed by Hardy and Higgins [2], is widely accepted. This hypothesis holds that the deposition of Aβ is the earliest event in AD progression, and that Aβ aggregation can trigger the tau-related pathologies of AD. The aggregation of Aβ is therefore a potential target for the diagnosis and treatment of AD. Aβ itself is considered a physiological by-product of cellular metabolism because it can be detected as a circulating protein in the cerebrospinal fluid and blood of healthy humans [3]; the physiological function of Aβ is summarized in a recent review by Panza *et al.* [4]. The 40-mer and 42-mer Aβ (Aβ40, Aβ42) proteins are produced from Aβ precursor protein (APP) [5,6]. The ability of Aβ42 to aggregate (i.e., oligomerize and fibrillize), and therefore to show neurotoxicity, is higher than that of Aβ40. Metastable oligomers of Aβ42 cause memory loss and synaptotoxicity, whereas the contribution of end-stage matured fibrils to the etiology of AD is smaller [7,8]. Aβ oligomers can exist in the equilibrium state, resulting in a heterogeneous mixture of various forms of intermediates by multiple pathways. Most Aβ42 oligomers are higher-order oligomers (24–700-mer) such as protofibrils (PFs) [9,10], Aβ-derived diffusible ligands [11], and amylospheroid [12], which are potently neurotoxic. Because there remain unanswered questions regarding which types of oligomers contribute the most to the pathogenesis of AD, developing multi-target inhibitors for these higher-order oligomers is imperative for making meaningful progress toward AD therapy. Furthermore, given the negative results in the recent clinical trials using Aβ-targeted drugs, some researchers are skeptic about the Aβ cascade. Meanwhile, functional food components and *Kampo* medicine with pleiotropic ability have gained attention. Especially, Kampo medicine has been long used for the symptomatic treatment of many diseases. Epidemiological studies have suggested that dietary habits could regulate the occurrence rate of AD. Several edible natural products have been characterized with effects such as anti-Aβ aggregation as well as anti-oxidative stress and anti-inflammation [13]. There have been a number of comprehensive papers including an encyclopedia and reviews on anti-Aβ aggregative polyphenols and flavonoids (e.g., chrysin, rutin, fisetin, resveratrol, and apigenin) [14,15,16,17,18,19,20,21,22,23]. However, the mechanisms underlying the anti-aggregative activity of most of these natural products remain to be elucidated.

The current review describes the polymerization mechanisms of Aβ42 and the strategy by which chemical agents such as flavonoids and terpenoids can control the aggregation or disaggregation of Aβ42. We focus on three structural features described under the following subsections: Section 3.1. catechol moiety, Section 3.2. planarity, and Section 3.3 carboxy group required for the activity of such chemical agents based on analysis using nuclear magnetic resonance (NMR) spectroscopy and mass spectrometry (MS). We also discuss the in vivo metabolism of these inhibitors, including blood–brain barrier permeability and brain–gut interaction, toward developing anti-AD drugs.

## 2. Nucleation-Dependent Polymerization Mechanism of Aβ42

### 2.1. Overview

**Figure 1:**
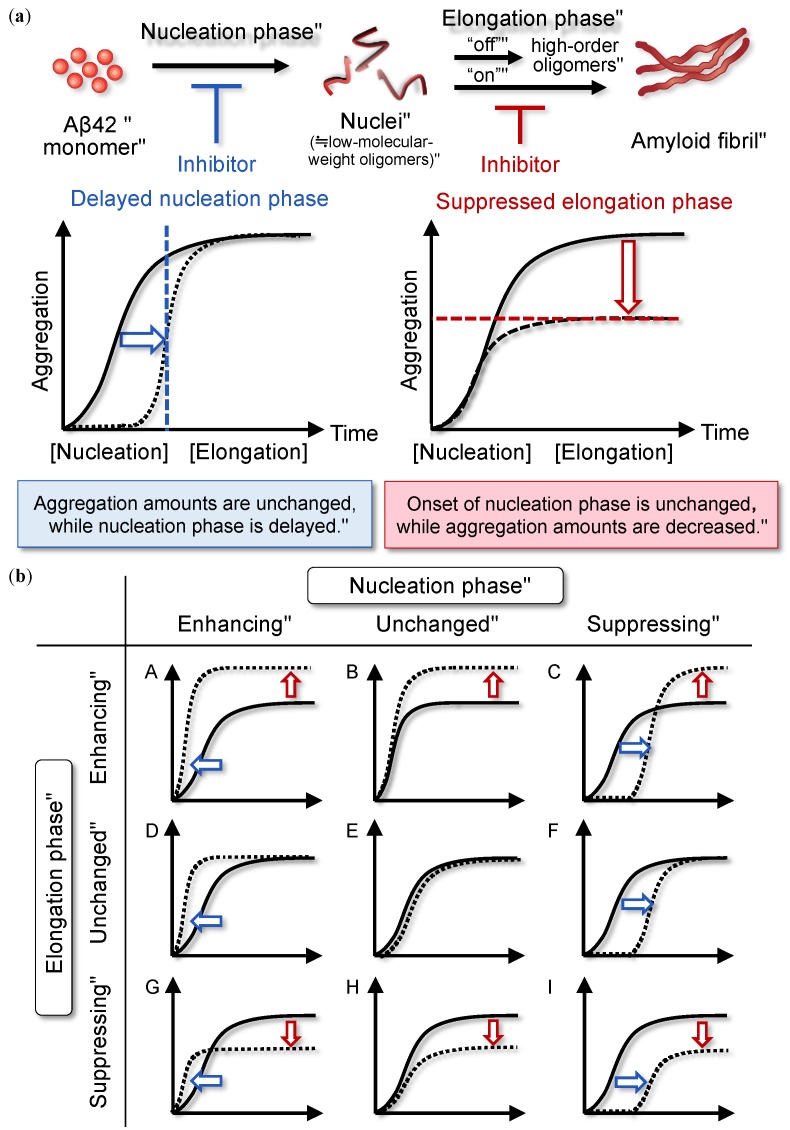
(**a**) Nucleation-dependent polymerization mechanism of amyloid β42 (Aβ42). The elongation phase is assumed to consist of “on”-pathway and “off”-pathway into fibrillization. (**b**) Categorization of nine types of modulators of Aβ42 aggregation focusing on the nucleation and elongation phases. In (**b**), only on-pathway route is indicated.

Naiki and coworkers proposed a nucleation-dependent polymerization mechanism that includes nucleation and elongation phases. This mechanism is currently accepted as an aggregation mechanism for Aβ42 in vitro [24]. During the nucleation phase, the monomeric Aβ42 gradually forms low-molecular-weight oligomers (i.e., “nuclei”) [25] before the elongation phase, during which each nucleus acts as a template and associates with monomers to initiate polymerization (Figure 1a). The elongation phase contains two pathways: on-pathway to move into fibrillization and off-pathway not to move into it. The higher-order oligomers mentioned above can exist between nuclei and amyloid fibril, though it remains controversial to which pathway each of the higher-order oligomers belong. Thereafter, depending on various factors including temperature, endogenous substances such as metal [26,27], lipid membrane [28], and apolipoprotein E [29], the amyloid products are roughly divided into two forms, namely well-defined cross-β-sheet amyloid fibrils and metastable high-order oligomers. In contrast to the neurotoxic high-order oligomers described above, fibrils as an end product are occasionally assumed to serve as an amyloid reservoir for confining toxic Aβ species [30]. Owing to the lower toxicity of fibrils, finding compounds that can convert oligomers into relatively inert fibrils, such as orcein-related compound O4, may be helpful for treating AD [31].

### 2.2. Classification of Aggregation Inhibitors

The nucleation phase involves the rate-limiting formation of oligomeric species associated with a lag time, in contrast with the elongation phase. The formation of oligomers that are kinetically competent to form fibrils occurs via a mechanism involving nucleated conformational conversion [32]. Because toxic oligomers could form kinetically competent nuclei, we consider the nucleation phase as a target for inhibition. Considering nine possible combinations of modulator patterns (Figure 1b), three types of modulators (“C,” “F,” and “I”) that delay the initiation of nuclei formation need to be focused on. It is important to systematically understand the rationale based on an organized analysis of how Aβ aggregation is suppressed.

Furthermore, another critical issue concerns how to prepare Aβ protein in a buffer solution when performing aggregation tests. In sodium dodecyl sulfate-polyacrylamide gel electrophoresis (SDS-PAGE), Aβ42 in a buffer solution produced predominantly not only a monomer but also a broad trimer or tetramer, which are assumed to be an SDS-induced artifact [33,34]. Since even fresh Aβ protein contains nuclei that are inevitably formed during HPLC purification and/or storage, Aβ42 likely aggregates very quickly to form larger nuclei. To evaluate Aβ42 aggregation, a sequential-type aggregation test using Aβ42 treated with 1,1,1,3,3,3-hexafluoro-2-propanol (HFIP), known as an α-helix inducer to dissociate β-sheet-contained nuclei [35], is useful. It must be noted that HFIP-treated Aβ42 forms a α-helix structure (but not a random structure) even before aggregation [36]. Indeed, Hiroaki and coworkers raised the problem that HFIP usage is not enough to dissolve Aβ42 nuclei into monomers (remaining as dimers) based on NMR studies [37].

## 3. Structural Features of Anti-Aβ42 Aggregative Compounds

### 3.1. Catechol-Type Flavonoid: Catechol Moiety

Most compounds with anti-aggregative properties against amyloidogenic proteins share a catechol moiety, which is also related to anti-oxidative stress, as exemplified by (+)-taxifolin, myricetin, quercetin, (+)-catechin, epigallocatechin gallate (EGCG), and rosmarinic acid [14,38,39,40]. Fink and coworkers first identified the possible significance of the *o*-quinone moiety of baicalein having the catechol structure in the A-ring for suppressing α-synuclein aggregation, which is responsible for Parkinson’s disease [41]. Kelly and coworkers also suggested the contribution of the oxidation product formed between EGCG and Aβ40 to the remodeling of preformed fibrils [42], but the structure of the oxidation product has not been identified. Lim and coworkers have comprehensively elucidated the mode of action underlying how unique compounds inhibit Aβ40 or Aβ42 aggregation by focusing on EGCG, myricetin, curcumin, nordihydroguaiaretic acid, rosmarinic acid, and ferulic acid [43,44,45,46]. To enhance the bioavailability of aggregation inhibitors, the linkage approach in order to design bivalent multifunctional compounds by conjugating cholesterol was adopted [47]. Notably, the existence of a catechol moiety in aminoisoflavones could be implicated in the differentiation of binding modes between aminoisoflavones and Aβ40 [45].

**Figure 2:**
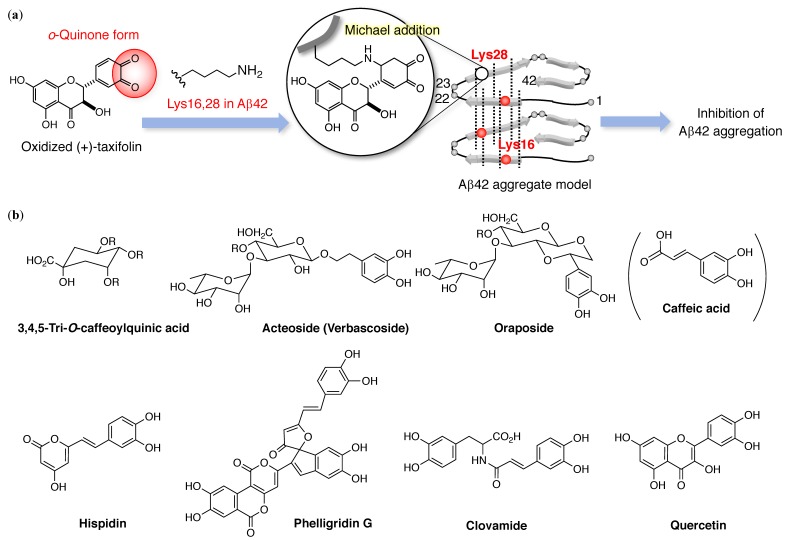
(**a**) Michael adduct of catechol-type flavonoids with Lys16 and 28 in Aβ42. (**b**) Catechol-type compounds from natural products by Shigemori and coworkers, which have anti-Aβ42 aggregation properties. R = caffeate.

Irie and coworkers demonstrated through systematic studies the role of the catechol moiety in preventing the aggregation of Aβ42, which has greater toxicity than Aβ40 aggregation. The *o*-quinone structure in the B-rings of (+)-taxifolin can target Lys16 and 28-Aβ42 via a Michael adduct, but not a Schiff base (Figure 2a) by targeting the elongation phase (categorized as “I” in Figure 1b) [38]. Ginex et al. validated the binding of (+)-taxifolin to the hydrophobic groove near Lys16 and Glu22, and suggested that this binding may proceed through the nucleophilic attack of the deprotonated amino group on the side chain of Lys16 [48]. Recently, the therapeutic potential of (+)-taxifolin was uncovered using a mouse model (Tg-SwDI mice) for cerebral amyloid angiopathy [49,50]; (+)-taxifolin was found to downregulate Aβ-related inflammation using laser speckle flowmetry to measure cerebral blood flow, as summarized in Table 1. This downregulation involves the modulation of the triggering receptor expressed on myeloid cell 2 (TREM2).

Shigemori and coworkers have carried out intensive studies on aggregation inhibitors of Aβ42 with a catechol moiety (Figure 2b). Caffeoylquinic acid is found in sweet potatoes, propolis, and coffee beans, and 3,4,5-tri-*O*-caffeoylquinic acid is a most potent analogue [51]. Acteoside [52] from *Orobanche minor* includes both caffeoyl acid and hydroxytyrosol, whereas oraposide [53] from *Orobanche minor*, hispidin and phelligridins [54] from *Inonotus obliquus*, and clovamide [55] from *Trifolium pratense* contain caffeoyl acid alone. Moreover, quercetin and its glycoside [56] are anti-oxidant flavonoids found in *Tamarix gallica*.

### 3.2. Non-Catechol-Type Flavonoid: Planarity

**Figure 3:**
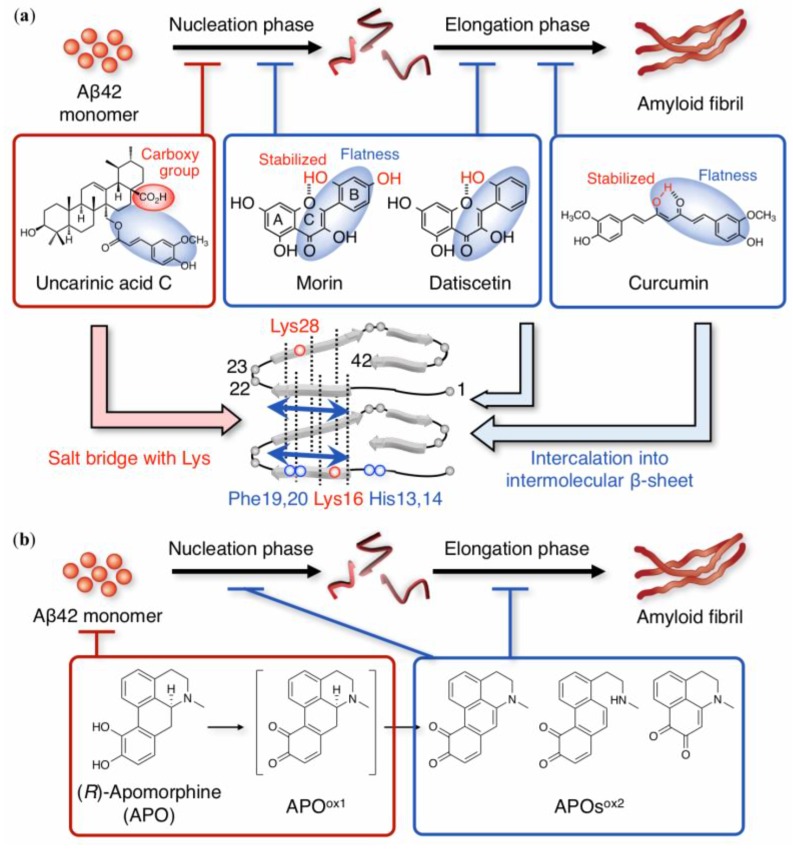
(**a**) Overview of aggregation inhibitors of non-catechol-type flavonoids. Morin and datiscetin inhibited both nucleation and elongation phases during the aggregation of Aβ42 by interacting with His13 and 14, and Phe19 and 20. (**b**) Mechanism of Aβ42 aggregation suppression by apomorphine (APO): APO is first autoxidized to the labile form (APO^ox1^), which includes *o*-quinone and has a biphenyl structure; APO^ox1^ forms the Michael adduct with Lys16 and 28 in monomeric Aβ42 during the nucleation phase, leading to the production of a secondary oxidized form (APOs^ox2^) during both nucleation and elongation phases. In (**a**,**b**), only the on-pathway route is indicated.

Several non-catechol-type compounds show potent inhibitory activities against Aβ42 aggregation. Solid-state NMR studies by Masuda et al. suggested a significant interaction between curcumin and the benzene rings of Phe19 and 20 in Aβ42 aggregates due to its intrinsic planarity and hydrophobicity, which allows it to interfere with the elongation phase [57] (Figure 3a). Richard et al. proposed the involvement of ε-viniferin glucoside, a non-catechol polyphenol, in the aromatic interaction between His13 and 14, and Phe19 and 20 of Aβ40 [58]. This interaction is related to the flatness derived from the aromaticity conjugated with an α,β-unsaturated ketone.

Morin and datiscetin are planar compounds similar to curcuminoids due to their aromatic B-ring and α,β-unsaturated ketone on the C-ring. Morin and datiscetin could affect both the nucleation and elongation phases (as “I”) [59], unlike curcumin (as “H”) [57]. Such a difference may be deduced from the difference in molecular sizes between flavonoid and curcuminoid. Intramolecular hydrogen bonding between the C-1 oxygen of the C-ring and the 2′-hydroxyl group of the B-ring can probably participate in stabilizing the flatness between the A-, B-, and C-rings. Indeed, the anti-aggregative ability of kaempferol, which lacks a 2′-hydroxyl group, is weaker than that of morin and datiscetin. Given that galangin, without the hydroxyl groups in the B-ring, is inactive, at least one hydroxyl group in the B-ring could be essential to its anti-aggregative ability [59]. ^1^H-^15^N heteronuclear multiple quantum coherence (HMQC) NMR with band-selective optimized-flip-angle short-transient (SOFAST) [60] suggested the common target residues His13 and 14, and Phe19 and 20 even in monomeric Aβ42 (Figure 3a). The involvement of these residues does not contradict the observation that intermolecular β-sheet regions (Gln15–Ala21 and Val24–Ile32) can form in Aβ42 aggregates (i.e., oligomers [61,62,63,64,65]), and that His13 and 14 act as coordinating sites of metal ions together with the ability to form the phenoxyl radical of Tyr10 and can trigger Aβ42 aggregation [66]. Regarding morin, there is one preclinical report on its attenuation of Aβ-related neuropathologies and cognitive impairment in APPswe/PS1dE9 mice [67] (Table 1).

Ano and coworkers reported on the prevention of several AD-related pathologies by iso-α-acids containing an α,β-unsaturated ketone, which are bitter components of beer, using a mouse model for AD [68]. Hisatsune and coworkers discovered the potential of β-alanyl-l-histidine (carnosine) in rescuing cognitive decline related to AD pathology [69]. Considering that carnosine, known as an endogenous anti-oxidant, is abundant in the brain, chicken meat may be a promising functional food, possibly because His residues may interfere with the regions rich in π electrons in Aβ42 aggregates.

(*R*)-Apomorphine, a dehydrated morphine, is a unique example whose target can vary depending on its oxidation states. Although (*R*)-apomorphine is known as a dopaminergic agonist in the treatment of Parkinson’s disease due to its potent anti-oxidant activity [70], we focused on the conjugated structure of (*R*)-apomorphine by autoxidation [71]. The extension of the conjugated system (isoquinoline skeleton) by autoxidation of the catechol moiety can enhance its planarity, leading to the retardation of further nuclei formation (Figure 3b). NMR studies showed that the first oxidized structure (APO^ox1^) of (*R*)-apomorphine can target Lys16 to form a Michael adduct in the nucleation phase. Coordinated structural changes can induce the generation of a secondary oxidized form (APO^ox2^), resulting in their intercalation into intermolecular β-sheet regions during the elongation phase (Figure 3b). Ohyagi and coworkers reported preclinical studies showing that (*R*)-apomorphine stimulated the degradation of intracellular Aβ [72] and mitigated the depositions of intracellular Aβ [73] in a 3xTg-AD mouse model (Table 1).

### 3.3. Triterpenoid Carboxy Acid: Carboxy Group

**Figure 4:**
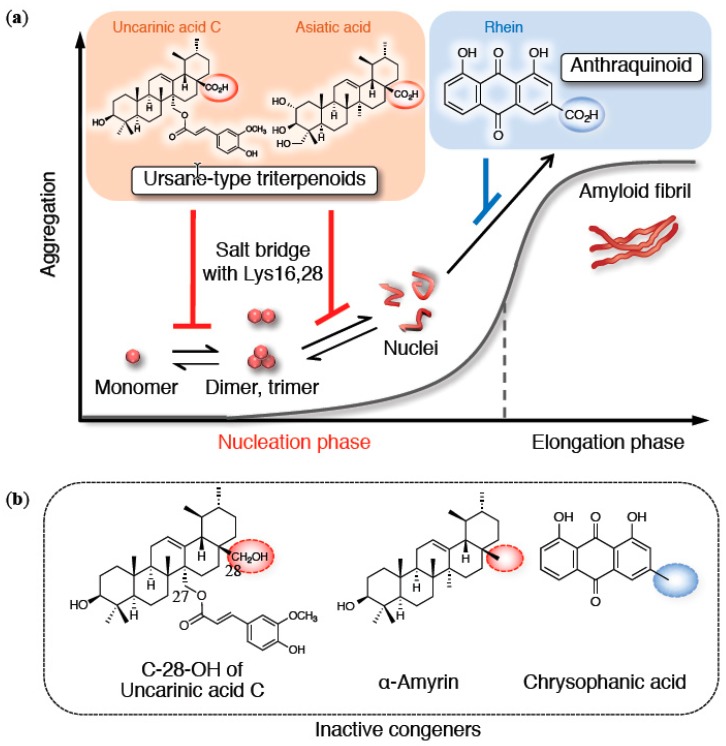
(**a**) Overview of aggregation inhibitors with ion bridge formation of their carboxy group with basic amino acids in Aβ42 (only on-pathway route is indicated). (**b**) Their inactive congeners as an ursane-type triterpenoid or an anthraquinoid.

As a third structural factor, the role of the carboxy group in anti-aggregative ability is well documented (e.g., α-d-mannosyl-glycerate [74] or flurbiprofen [75]). Actually, we validated the potential of uncarinic acids A–D such as triterpenoid carboxy acids [76] from *Uncaria rhynchophylla*, also known as *chotoko* (a *shoyaku*), which are specific inhibitors of the nucleation phase. Uncarinic acids A–D are categorized as “F” or “I” in the inhibition patterns of Figure 2b. The structure–activity studies of uncarinic acid C (Figure 4a), which is able to prevent Aβ42-associated neurotoxicity, showed both a C-27 ferulate and a C-28 carboxylic acid group to be important for its inhibitory activities. According to SOFAST HMQC in NMR and ion mobility–mass spectrometry (IM-MS) combined with native ionization techniques, these inhibitor activities could be associated with the formation of a salt bridge with Lys16, resulting in the prevention of dimer or trimer formation, which can be a possible minimum unit of toxic oligomers (2 or 3 x *n*-mer) [36]. The flatness derived from C-27 ferulate may also participate in the interaction with either monomeric Aβ42 or its aggregates.

Ion mobility-mass spectrometry (IM-MS) is a powerful tool for measuring metastable oligomers to detect individual ions dependent on molecular size and drift time because of the inherent tendency of native oligomers to form structurally heterogeneous assemblies with the same mass-to-charge ratio (*m*/*z*) [77,78,79]; IM-MS can clearly separate these assemblies despite their identical *m*/*z* ratios in place of conventional techniques such as SDS-PAGE, size exclusion chromatography, and photo-induced cross-linking, as reviewed in Reference [80]. The unnecessity of using organic solvents that disrupt the non-covalent interactions within the Aβ oligomers is one of the merits of this tool. Intriguingly, the use of SDS causes the overestimation or underestimation of the amount of oligomer because SDS likely induces or interferes with Aβ oligomerization [81,82]. Comparing the arrival time distribution of each signal (*z*/*n*; *z* denotes charge, *n* denotes the number of oligomers), peaks corresponding to oligomeric orders can be assigned to the series of multivalent ions at least up to 24-mer [83]. IM-MS was also applied to the screening method of aggregation inhibitors to classify small molecules with anti-aggregation of Aβ40 into specific (e.g., EGCG), non-specific (e.g., tramiprosate), colloidal (e.g., congo red), or non-interacting (e.g., hemin) [79].

To further investigate the contribution of the carbon skeleton with a carboxy group, asiatic acid (ursane-type triterpenoid skeleton) from *Centella asiatica* in gotu kola and rhein (anthraquinoid skeleton) from *Rheum palmatum* in daio were selected together with α-amyrin and chrysophanic acid as the corresponding comparators [36] (Figure 4b). Asiatic acid retarded the nucleation of Aβ42, also focusing on the dimer or trimer, despite lacking a ferulate moiety. In contrast, such activity was diminished in α-amyrin. Maslinic acid, a similar triterpenoic acid, has been shown to protect against apoptosis induced by Aβ42 [84]. Rhein, categorized as either “H” or “I,” inhibited the elongation phase more than the nucleation phase because of the prioritized planarity originating from its anthraquinone skeleton. Actually, chrysophanic acid prevented the elongation as “H,” albeit with a slightly weaker inhibition of nucleation than α-amyrin. These findings validate the preferable effects of the carboxy groups of triterpenoids in attenuating toxic nuclei-driven oligomerization.

## 4. In Vivo Metabolism of Anti-Aβ42 Aggregative Compounds

Despite the efforts of many researchers, no Aβ aggregation inhibitors that have ever been listed in clinical trials can completely meet the demands of pharmaceutical manufacturers [4]. Although sodium oligomannurarate (GV-971, a marine glycan-derived drug; ClinicalTrials.gov Identifier: NCT02293915) from a Chinese company currently in a Phase III trial is attracting attention (Xiao, S. et al. Phase 3 clinical trial for a novel and multi-targeted oligosaccharide in patients with mild-moderate AD in China (abstract OC3), presented at the 11th Clinical Trials on Alzheimer’s Disease, Barcelona, Spain, 2018), most principal clinical studies of natural products have failed. These failed studies include EGCG from green tea (ClinicalTrials.gov Identifier: NCT00951834; trial results were not published, but see Reference [85]), scyllo-inositol from coconut palm (e.g., ClinicalTrials.gov Identifier: NCT01735630) [86], and curcumin from turmeric (e.g., ClinicalTrials.gov Identifier: NCT00099710) [87] (Table 2). According to the paper by Nelson et al. [88], curcumin has never shown effective outcomes in randomized, placebo-controlled clinical trials [89]. This is thought to be due to its instability and low bioavailability, as curcumin abundantly exists as a glucuronide and sulfate conjugates of metabolized or degraded forms in blood and excretions [88]. Considering the p*K*_a_ values (8.5–10.4) of curcumin, the transformation into the phenolate form in water can easily lead to its degradation to vanillin, ferulic acid, and feruloylmethane at neutral and alkaline pH. Accordingly, the amount of intact curcumin in the brain is too small to be detected.

Meanwhile, curcumin may affect the composition of the gut microbiota, possibly leading to an alteration in brain-gut interaction [90]. There is a growing interest in the preventive role of intestinal microbiota against several lifestyle diseases including neurodegenerative disorders. Minter et al. identified the therapeutic potential of antibiotic-induced perturbations of gut microbial diversity in the pathology of AD [91]. Several studies on the metabolites of dietary fibers by intestinal microbiota in the colon have identified short-chain fatty acids (less than C5) such as valeic acid, isovaleric acid, isobutyric acid, butyric acid, propionic acid, acetic acid, and formic acid, which serve as substrates for energy resources [92,93]. Actually, these short-chain fatty acids attenuated the oligomer formation of Aβ40 and Aβ42 in vitro [94]. In contrast, there is an opposing report that the absence of gut microbiota reduced Aβ-related pathology in a mouse model for AD [95]. Further studies will be required to resolve these controversial issues regarding what kinds of microbiota can make major contributions to AD and other neurodegenerative disorders.

In general, it is reported that 0~60% of flavonoids are absorbed in the small intestinal and their half-lives are between 2 and 28 h [96]. In the following metabolism, absorbed flavonoids can go through first pass Phase II, thereby transforming into methylated, sulfated, and glucuronated forms in plasma. These alternations may mutually affect the activity of cytochrome P450 [97]. In positron emission tomography studies by Kan and coworkers, a small amount (<1%) of intravenous-injected nobiletin was detected [98]. One could argue about what percentage of administered compounds is needed for therapeutic effects in the brain. Too many doses for administration to increase uptake may lead to unknown side effects in digestive organs. Recently, there are two reports that support underappreciated thinking that brain-targeted compounds do not always need to go through the blood–brain barrier to exert an effect. Cote et al. [99] and Duca et al. [100]. suggested a new role of resveratrol and metformin in duodenal energy sensing in the gut-brain-liver communication axis.

## 5. Conclusions and Perspective

To conclude, we offer three structural factors required for suppressing Aβ42 aggregation including oligomerization, thereby paving the way for future research to design disease-modifying drugs such as toxic oligomer-targeted inhibitors. Moreover, this knowledge could be useful for predicting whether natural products have the ability to inhibit Aβ42 aggregation. Essentially, it should be noted that these factors may also determine whether the nucleation or elongation phases are targeted. The most-targeted amino acid residues in Aβ42 aggregates include Lys16 and 28 for Michael addition and ion-bridge formation, and His13 and 14 as well as Phe19 and 20 for intercalation. Most of these residues are localized in β-sheet regions [62]. Recently, prion-like cell-to-cell transmission of Aβ oligomers was identified as one potential mechanism by which the toxicity of Aβ oligomers could spread [101]. The impact of the metabolites of test compounds together with their pharmacokinetics, blood-brain barrier permeability, and effective conjugate forms must be thus explored through further cell culture or in vivo experiments.

## Figures and Tables

**Table 1:** Preclinical studies of compounds in this review using Alzheimer’s disease (AD) model mice.

Drug	Mouse	Aβ Pathology	Memory Loss	Remarks	Ref.
(+)-Taxifolin	Tg-SwDI	SP, oligomer decreased	Improved (MWM)	Impaired CBF restored	[49,50]
Morin	APPswe/PS1dE9	SP, Aβ production decreased	Improved (MWM)	Tau-P decreased	[67]
(*R*)-Apomorphine	3xTg-AD	SP, intracellular Aβ decreased	Improved (MWM)	Tau-P decreased	[72,73]

Abbreviations: CBF, cerebral blood flow; MWM, Morris water maze; SP, senile plaque; Tau-P, tau phosphorylation.

**Table 2:** Failed clinical studies of natural products targeting Aβ aggregation.

Drug	Patient	Enrollment	Phase	Outcome	ClinicalTrials ID
EGCG	Early AD	21	II, III	Insufficient efficacy	NCT00951834
*scyllo*-inositol (ELND005)	Moderate to severe AD	350	II	Insufficient efficacy	NCT01735630
Curcumin C3 Complex	Mild to moderate AD	33	II	Insufficient efficacy	NCT00099710

Abbreviations: AD, Alzheimer’s disease; EGCG, epigallocatechin gallate.
